# Epitaxial graphene for quantum resistance metrology

**DOI:** 10.1088/1681-7575/aacd23

**Published:** 2018

**Authors:** Mattias Kruskopf, Randolph E Elmquist

**Affiliations:** 1 National Institute of Standards and Technology, Fundamental Electrical Measurements, 100 Bureau Drive, Gaithersburg, MD, United States of America; 2 University of Maryland, Joint Quantum Institute, College Park, MD, United States of America

**Keywords:** epitaxial graphene, quantum Hall effect, quantum resistance metrology

## Abstract

Graphene-based quantised Hall resistance standards promise high precision for the unit ohm under less exclusive measurement conditions, enabling the use of compact measurement systems. To meet the requirements of metrological applications, national metrology institutes developed large-area monolayer graphene growth methods for uniform material properties and optimized device fabrication techniques. Precision measurements of the quantized Hall resistance showing the advantage of graphene over GaAs-based resistance standards demonstrate the remarkable achievements realized by the research community. This work provides an overview over the state-of-the-art technologies in this field.

## 1. Introduction

Quantum standards in the new SI promise to provide ‘access for all’ primary units based on internationally recognized physical realizations. The laser, for example, has made standards of length inexpensive and accurate in terms of another easily accessed quantum standard, the global positioning system (GPS) broadcast [1]. Electrical standards based on the quantum Hall effect (QHE) have succeeded in yielding unprecedented consistency and accuracy, but have lagged at reaching the broad user level of benchtop metrology due to the high costs and the complexity of the systems. Thus, major national metrology institutes (NMIs) are focused on creating graphene-based quantised Hall resistance (QHR) standards that operate above 2.5 K, below 5 T, and with cur rents an order of magnitude larger than used in present-day GaAs QHR standards. This effort addresses basic needs of the metrology community and advances graphene for other uses by developing techniques to control graphene’s electronic characteristics.

The emergence of epitaxial graphene (EG), grown with intrinsic structural alignment on SiC crystals, is allowing the metrology community to realize the extraordinary precision of the new QHR standard more efficiently. This material is produced by sublimation of Si from nearly defect-free hexagonal SiC (0001) wafers at temperatures up to 2000 °C [2–4]. With extended high temperature annealing, crystalline SiC (0001) forms a graphitic surface with Bernal-stacked layers [5]. The epitaxial layer closest to the substrate lacks the electronic properties of graphene due to strong covalent bonds, and is described as the buffer layer (BL). The first layer of epitaxial graphene (EG) is formed as a new BL grows, breaking the bonds between the substrate and the EG layer [6]. This EG layer is conductive only in two dimensions with the band structure of graphite as first calculated by Wallace at the National Research Council of Canada in 1947 [7]. Multiple symmetries are present in the 2D honeycomb lattice. This produces the electronic analog of massless, relativistic charge carriers which can be either electrons or holes [8].

The observation of the QHE at room temperature in graphene has attracted intense interest in physics and metrology communities [9, 10]. In 2010, Tzalenchuk and co-authors from the National Physical Laboratory (NPL) in London and university groups in Sweden, Italy and the UK observed EG devices having contacts of low-resistance (≈1.5 Ω) and Hall quantization uncertainty of 3 n Ω Ω^−1^at 0.3 K [11]. In 2011, the same authors collaborated with scientists from the International Bureau of Weights and Measures (BIPM) in an improved measurement by direct comparison of an EG QHR device with two GaAs standards obtained from the Physikalisch-Technische Bundesanstalt (PTB) and Laboratoires D’electronique Philips (LEP). The weighted mean of results using a symmetric cryogenic current comparator bridge with source-drain currents between 35 μA to 100 μA was (*R*GaAs/AlGaAs – *R*Graphene)/(*h*/2*e*^2^) = (−4.7 ± 8.6)×10^−11^.

Specialized molecular-beam epitaxy (MBE) systems had been necessary for the growth of GaAs/AlGaAs heterostructures having mobilities of 15 m^2^ V^−1^ s^−1^ to 40 m^2^ V^−1^ s^−1^ for quantised resistance plateaus of width 1 T to 2 T [12]. The standardization of QHR device fabrication by National Measurement Institutes (NMIs) using EG appeared to offer much lower technical barriers than the continued production of GaAs-based standards, at least in the initial EG wave of research that began in 2010. Characterization of these early devices revealed that mobilities of order 0.4 m^2^ V^−1^ s^−1^ to 0.7 m^2^ V^−1^ s^−1^ could provide quantised *i* = 2 plateaus which can extend from low magnetic flux densities of *B* ≈ 2 T to very high values of *B* ⩾ 15 T with proper control of the carrier concentration. Several papers from the NPL collaboration have attributed this plateau-broadening effect to charge transfer at the substrate/graphene interface, which extends the range of the plateau to very high magnetic field [13, 14]. Both the PTB and the National Institute of Standards and Technology (NIST) established systems as a basis for EG growth using rf-inductive or ac-resistive heaters, and several NMI groups formed partnerships with researchers at universities, with the largest being the European joint research project GraphOhm [15].

## 2. Monolayer epitaxial graphene

**Figure 1.**
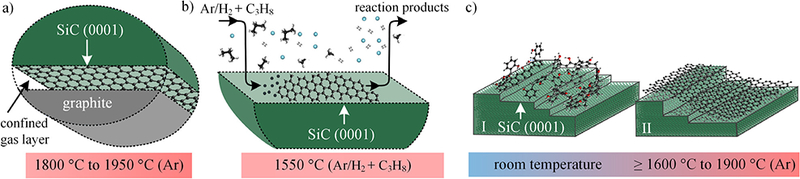
Large-area growth methods of epitaxial graphene on SiC used at NMIs. (a) In face-to-graphite (FTG) growth, a confined gas layer between the SiC substrate and a polished graphite disk ensures high partial pressures of sublimating species and allows for controlled growth at high process temperatures. (b) In chemical vapor deposition (CVD), the carbon rich precursor (C_3_H_8_) and the carrier gas (Ar/H_2_) are used to control the growth conditions at relatively low temperatures. (c) Using polymer-assisted sublimation growth (PASG), nucleation is enhanced during the early growth stages of the buffer layer such that the bunching of atomic planes is suppressed.

SiC surfaces decompose by sublimation at high temperature in a vacuum or a background of non-reactive gas, forming Si-rich vapor and atomic carbon that remains on the substrate. Surface carbon assembles into hexagonally bonded EG sheets that can be relatively free of lattice defects depending on growth conditions and can be imaged using surface-science techniques. Figure 1 illustrates three growth methods for large-area epitaxial graphene growth that are used by NMIs. All processes apply a background pressure of argon close to atmospheric pressure which directly affects the partial pressures of sublimating species (e.g. Si, Si_2_C, SiC_2_) by reducing their mean free path length compared to vacuum. This creates a near-equilibrium growth environment with higher partial pressures near the surface, which reduces the defect density in the graphene layer significantly [16–18]. The face-to-graphite growth (FTG) method applied at the NIST (figure 1(a)) follows this route by creating a confined gas layer of sublimating species in the gap between a polished glassy carbon disk and downwards facing SiC (0001) substrate [19–21]. The Laboratoire National de Métrologie et d’Essais (LNE) and a consortium of research institutes developed a successful way to realize controlled graphene growth on SiC by chemical vapor deposition (CVD) using a carbon rich propane precursor (C_3_H_8_) in an Ar/H_2_ ambient (figure 1(b)) [22–24]. The polymer-assisted sublimation growth (PASG) developed at the PTB enhances the nucleation of the buffer layer in the early growth stages which allows for the conservation of sub-nanometer SiC terrace heights throughout EG formation ((figure 1(c)) [25, 26]. Each of these techniques suppresses the formation of bilayer graphene domains as well as of high step edges allowing for the fabrication of high-mobility QHE devices.

### 2.1. Structural characterization of the EG layer

Bilayer domains and high step edges are known to result in conductance anisotropies that can lead to a breakdown of the quantum Hall effect [27, 28]. Narrow bilayer regions tend to form along step-edge facets where the edges of many SiC (0001) atomic layers are exposed through ‘step bunching’ [29, 30]. Potential reasons for the negative impact of step edges are variations of the doping level, scattering centers, and strain due to a local detachment of the graphene layer at the terraces edges of the substrate [31–34]. To identify bilayers, fast and non-destructive characterization methods of the graphene material and devices are an essential part in the fabrication process.

**Figure 2.**
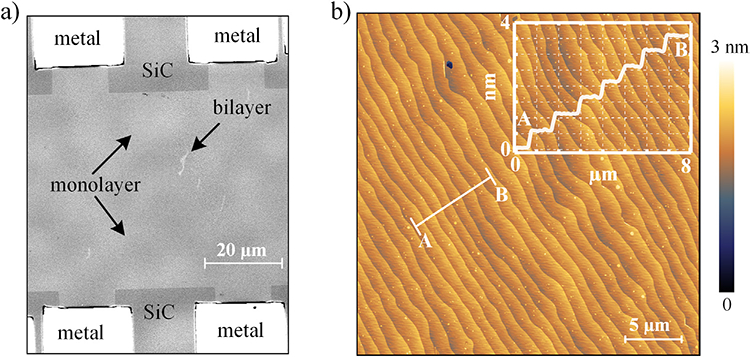
(a) The CLSM image of a graphene QHE device shows the metal contacts (white), the structured graphene monolayer (grey) and the etched SiC (dark grey). Bilayer patches are identified by small, light grey patches. (b) Typical surface morphology of epitaxial monolayer graphene on 4H-SiC (0001) grown by a combined approach using face-to-graphite (FTG) and polymer-assisted sublimation growth (PASG). The inset shows the regular terrace structure with step heights of 0.50 nm for the angle-corrected profile line.

Topographic imaging by atomic force microscopy (AFM) can reveal the SiC terraces, however the identification of EG domains is far from straight-forward using AFM alone. Confocal laser scanning microscopy (CLSM) [35] and optical microscopy using digital contrast enhancement [36] are excellent quantitative techniques to distinguish layer inhomogeneities. CLSM has a lateral resolution somewhat beyond the optical diffraction limit and achieves improved depth-of-field by combining images at different focal planes, allowing scaling to larger imaging areas, as demonstrated by the CLSM image of a graphene device in figure 2(a)). Figure 2(b) shows the AFM image of a typical terrace structure of epitaxial monolayer graphene grown by face-to-graphite and polymer-assisted growth which allow for the conservation of exceptionally low steps ⩽ 0.75 nm on the centimeter-scale samples with minimum resistance aniso tropy [25, 34, 37].

**Figure 3.**
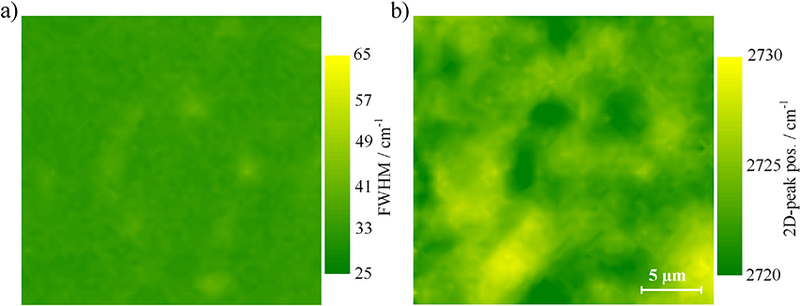
Raman area mappings of the 2D-peak (G’) characteristics of epitaxial monolayer graphene on 4H-SiC (0001) using the FTG and PASG growth methods show a typical distribution of (a) the FWHM with a mean of 33.2 cm^−1^ ± 1.4 cm^−1^ and (b) the peak position with a mean of 2723.3 cm^−1^ ± 1.8 cm^−1^ across the surface.

Raman spectroscopy is a fast and non-destructive tool to identify monolayer graphene, whose fingerprint in the Raman spectrum is a symmetric G’ peak (2D band) at ≈ 2700 cm^−1^ that can be fitted by a single Lorentzian. In addition, the full-width-half-maximum (FWHM) of the 2D(G’) peak is broadened by stress and defects [38–41]. The Raman measurements in figures 3(a) and (b) show typical characteristics of an epitaxial graphene monolayer on SiC (0001) with a FWHM of 33.2 cm^−1^ ± 1.4 cm^−1^ and a peak position of 2723.3 cm^−1^ ± 1.8 cm^−1^, produced using the combined methods of FTG and PASG as inhibitors of growth. In comparison, graphene epitaxy on SiC by CVD has a lower peak position around 2695 cm^−1^ [42, 43]. This is close to the peak position of exfoliated and hydrogen intercalated epitaxial graphene and indicates reduced graphene/substrate interactions when using the ‘top-down’ CVD growth process [41, 44].

### 2.2. Device fabrication and electrical characterization

**Figure 4.**
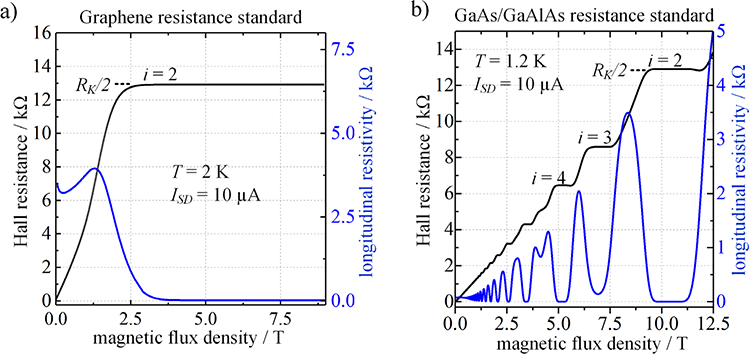
Resistance quantization in the 2DEG of graphene and GaAs/GaAlAs. (a) In the case of epitaxial graphene at a charge carrier density of about *n ≈* 1.5 × 10^11^ cm^−2^ the *i* = 2 plateau becomes accessible at relatively low magnetic flux densities of *B* ⩾ 3 T once the longitudinal resistivity vanishes. For resistance metrology, the *i* = 2 plateau with the value of *R*_H_ = *R*_K_/2 is used as a reference for the unit ohm. (b) For GaAs/GaAlAs resistance standards, a series of Hall resistance plateaus develop within the typical range of magnetic flux densities (0T ⩽ *B* ⩽ 15 T) with the *i* = 2 plateau starting around *B* = 10 T for typically chosen carrier concentrations slightly below 5 × 10^11^ cm^−2^.

Starting from measurements in the early days of graphene research, exfoliated graphene samples showed robust quantization as predicted for the chiral Dirac-fermion electronic carriers, with symmetric behavior for either electrons or holes. In single-layer graphene, the lowest LL is unique in that it is occupied by both types of carrier, with two degenerate hole states and two for electrons [45]. All other LLs have four-fold degeneracy for one or the other carrier, thus yielding the anomalous half-integer QHE plateau sequence (*ρ*_*xy*_ = *h*/2*e*^2^, *h*/6*e*^2^, …). While the *i* = 6 plateau is usually not visible in the case of low charge carrier density devices, the *i* = 2 plateau is exceptionally broad as shown in (figure 4(a)). This is due to the before-mentioned charge transfer mechanism at the substrate/graphene interface for increasing magnetic flux density [13, 14].

**Figure 5.**
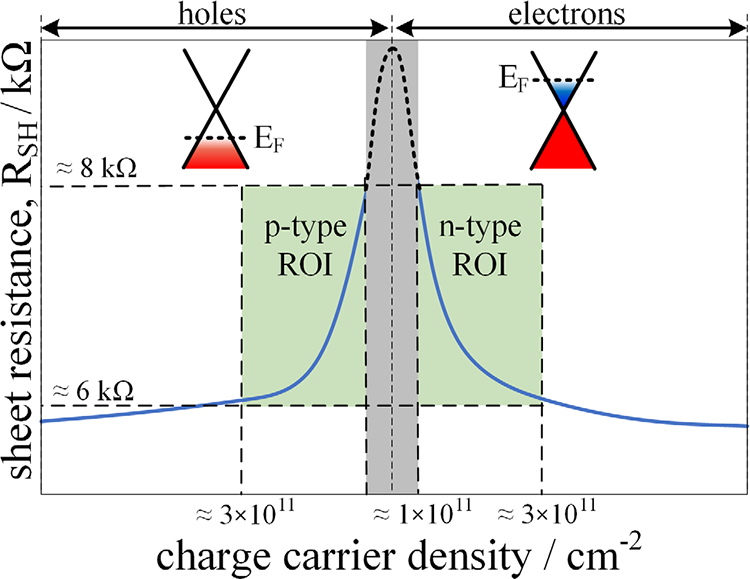
Sheet resistance versus carrier density of epitaxial graphene samples and room temperature best-practice values defining the region of interest (ROI) for quantum resistance metrology.

The transport characteristics of a graphene sample are best described by the relation of the charge carrier density *n* = 1/*q*μ*R*_SH_ with respect to the sheet resistance *R*_SH_ and the charge carrier mobility μ, where *q* is the charge. The control and stabilization of these electronic properties are one of the major remaining challenges for the implementation of graphene for quantum resistance metrology [46–48]. The relationship between *R*_SH_ and *n* shown in figure 5 allows for a straightforward way to approximate the carrier density of a graphene device without performing Hall measurements under the assumption of homogenous and reproducible device quality. By measuring the room temperature sheet resistance *R*_SH_ = *R*_*xx*_ × *w/l*, where *R*_*xx*_ is the resistance between two neighboring contacts, *w* is the width of the device and *l* is the distance between the two contacts one can estimate a charge carrier density of 3 × 10^11^ cm^−2^ ⩾ *n* ⩾ 1 × 10^11^ cm^−2^ for typically 8 kΩ ⩾ *R*_SH_ ⩾ 6 kΩ. Note that this method allows for fast pre-characterization of graphene QHE devices at room temperature but does not replace the precise determination of *n* from Hall measurements. For low values of *n*, the mobility, *μ* increases strongly in homogeneous devices. For *n* ≈ 0, the sheet resistance reaches a maximum value where both holes and electrons are present in equal concentration, forming ‘puddles’ of either type as determined by local potentials from defects and trapped charge [49, 50].

In semiconductor quantum Hall systems, the plateaus in *ρ*_*xy*_ are centered at magnetic field values *B*_c_ = *enR*_H_, where *n* is the density of charge carriers and *R*_H_ is equal to *h*/*ie*^2^ with the quantum filling factor *i* taking on integer values. Layer-by-layer growth of GaAs–GaAlAs heterostructures used in metrology requires molecular beam epitaxy, with careful control of doping layers brought about by specific adjustment of atomic concentrations. To maintain high carrier mobility the 2D electronic carriers are physically separated from the layer of positively ionized donor atoms by modulation doping [12], which allows the carrier density to be fixed at useful levels. Thus, the different band gaps at the interface between GaAs and GaAlAs create an electrostatic potential well. The width of the plateau for a given quantum number *i* is related to the level of disorder in the device, which causes broadening of the Landau levels (LLs). Figure 4(b) shows the resistance plateaus of a GaAs/GaAlAs resistance. Typically, only the *i* = 4 and *i* = 2 plateaus have sufficiently low dissipation to yield the precise quantization needed for resistance metrology, but the specific fabrication processes can be tuned to meet the requirements for quantised Hall resistance standards [51, 52].

National metrology institutes have adopted several methods to control the transport properties of EG to compensate for excess electron doping from the covalent BL bonds such that the *i* = 2 plateau appears below 5 T, as shown in figure 4(a). The devices employed at the NPL, LNE, PTB and the Centre for Metrology and Accreditation (MIKES) of Finland utilize two different gating methods developed at the Chalmers University. The first technique requires the spin coating of a photosensitive polymer and a spacer layer which then creates an electrostatic potential upon exposure to UV-light [53]. The second method for tuning the carrier density in epitaxial graphene is via electrostatic potential gating with ions produced by corona discharge [54]. A third method, developed by the NIST group, produces p-type molecular doping from nitric acid or aqua regia used as a gold etchant in the device fabrication process [6]. These present-day methods are insufficient for the ideal QHR standard, however, with reports of poor long-term stability [55–57].

Figure 5 shows the symmetric relationship of the sheet resistance versus the charge carrier density for p-type and *n*-type conductivity. For lower charge carrier densities of |*n*| < 1 × 10^11^ cm^−2^ (grey shade) the critical current drops significantly as electron/hole puddles appear, while for |*n*| > 3 × 10^11^ cm^−2^ (white region) the resistance plateau is shifted to relatively high magnetic flux densities. The green shade region indicates the region of interest (ROI) for resistance metrology with 1 × 10^11^ cm^−2^ ⩽ |*n*| ⩽ 3 × 10^11^ cm^−2^ and a typical room temperature sheet resistance of 6 kΩ ⩽ *R*_SH_ ⩽ 8 kΩ.

Many of the difficulties that affect semiconductor hetero-structure QHR devices have analogs for epitaxial graphene and graphene devices in general. One example is the requirement for metallic ohmic contacts with a low and stable contact resistance [12, 58]. The metal-graphene electronic coupling strength [59] is dependent on the physical separation and the height of the potential barrier between the metal and the 2D layer, and metal-interface bonding is weak for pristine graphene since no dangling bonds are present. The coupling strength is dependent on van der Waals forces unless defects are intentionally introduced, for example by prior plasma etching [60]. Strongly interacting metals, notably Pd and Ni [61, 59], may increase the surface and edge interactions and reduce the metal-graphene contact resistance.

**Figure 6.**
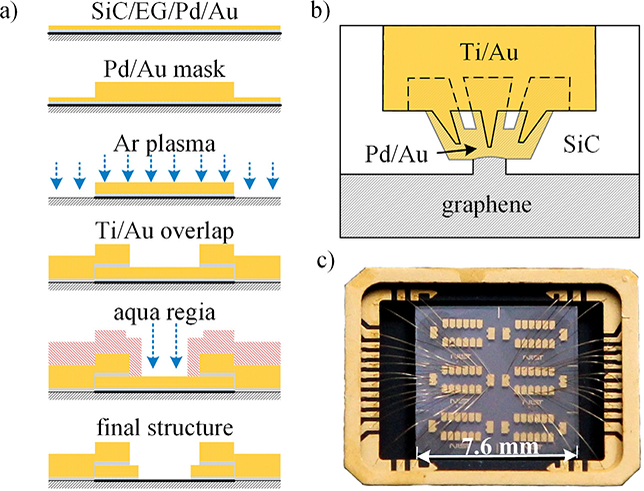
Graphene devices and fabrication techniques for quantum resistance metrology. (a) Devices are fabricated using a contaminant-free, six-step process by photolithography (from the top to the bottom). (b) The EG/metal contact is realized by Pd/Au that is mechanically supported by overlapping Ti/Au fingers. (c) The photograph shows two measurement-ready graphene QHE devices (2.2 mm × 0.4 mm) mounted in 32-pin chip carrier.

Figure 6(a) describes the device fabrication process applied at NIST that is optimized to shield the graphene from lithographic residues and to produce a contaminant-free EG/metal contact [21, 62]. This shielding is accomplished by depositing a thin Pd/Au layer which forms the graphene/metal contact in the first step and likewise protects the graphene throughout the process. The graphene Hall-bar features are then structured using argon plasma and an Au metal mask that covers the contact area as well as the active Hall bar area. Figure 6(b) shows the contact geometry used at NIST. While the contact geometry allows for great flexibility, extra care needs to be taken to improve physical adhesion of the bond pads and the EG/metal contacts. To improve adhesion, the graphene layer is removed to expose the bare SiC substrate before the Ti/Au bond pads are deposited which partially overlap the Pd/Au contacts. These bond pads support conventional wire-bonding and provide a firm anchor for the connected contact area. Windows or meandered graphene edges can improve the side-contacting capability of the sheet which is reported to reduce the contact resistances [63]. In the last step of device fabrication, aqua regia is applied to selectively remove the Pd/Au layer covering the Hall bar region using a photoresist mask. Figure 6(c) shows two final graphene QHE devices after wire bonding to a 32-pin chip carrier.

## 3. QHR parameter space

**Figure 7.**
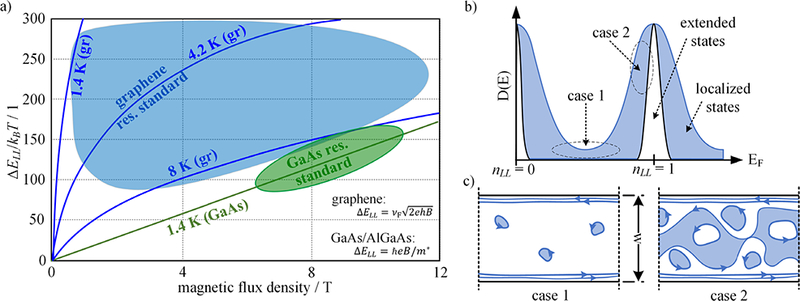
(a) The energy splitting of the LLs forming the *i* = 2 plateau shows a square-root magnetic field dependence for graphene (gr) and a linear dependence for GaAs/AlGaAs (GaAs). The individual curves show the ratio of the field dependent energy splitting with respect to different thermal energies *k*_B_*T*. (b) and (c) Between the two LLs *n*_LL_ = 0 and *n*_LL_ = 1, the density of electronic states D(E) shows minima with only localized states (case 1). Only in case 1, the dissipation-less, one-dimensional (1D) edge channels are isolated from each other and enable resistance quantization. As a function of the Fermi energy *E*_F_ extended states develop in the sample when the D(E) approaches the maxima e.g. at *n*_LL_ = 1 (case 2) leading to a breakdown of the QHE.

Figure 7 demonstrates the temperature and field dependence of the energy splitting of the LLs, Δ*E*_LL_ forming the *i =* 2 plateau for single-layer graphene (1LG) [45, 64] and GaAs/AlGaAs (GaAs) [65] with respect to the thermal energy, *k*_B_*T*. The large energy splitting is a result of the square-root magnetic field dependence and causes a rapid widening of the energy gap forming the *R*_K_/2 resistance plateau even at low values of *B*. If the ratio Δ*E*_LL_/*k*_B_*T* ≈ 150 of typical GaAs-based resistance standards operating at a temperature of 1.4 K and *B* ≈ 10 T is taken as the reference value, one can expect much lower magnetic flux densities and higher temperatures for the measurements when using graphene [66].

Considering a measurement temperature of a typical closed-cycle table-top cryostat operating between 2.8 K and 4.2 K, QHR standards could be maintained at magnetic flux densities between 1 T and 4 T for graphene as indicated by the blue shaded area in figure 7(a). Reasons for why the energy splitting of the LLs must be at least 100 times larger than the thermal energy include electron heating at higher currents and inhomogeneities in the charge carrier density of the sample [49, 67]. Figure 7(b) shows the density of states between the two LLs *n*_LL_ = 0 and *n*_LL_ = 1 forming the *i* = 2 resistance plateau in graphene as a function of the Fermi energy *E*_F_ [68]. The best resistance quantization is reached when all states inside the sample are localized and the high and low potential edge channels of the Hall bar with the width *w* are isolated from each other (see case 1, figure 7(c)). Once extended states develop (see case 2, figure 7(c)), resistance quantization breaks down. Compared to GaAs and bilayer graphene, the band structure of monolayer graphene does not contain a second conduction band that could result in parallel conducting channels at elevated thermal energy [69] which enables traces of the QHE to be preserved even at room temperature in some ultra-clean samples at high magnetic flux densities [9].

Inhomogeneous magnetic fields lead to a breakdown of the QHE in semiconductor systems due to the relatively narrow LL energy gaps and the fixed number of electronic carriers that can contribute to 2D conduction. It is easy to see that the effect of local inhomogeneity in B is like inhomogeneity in the carrier density, since both tend to broaden the LLs and thus reduce the energy gaps. A related source of disorder in graphene QHE systems stems from deformations in the lattice, whether spontaneous (corrugation, ripples) or by the interaction with the substrate, which both cause strain that can create effective inhomogeneous magnetic fields [70]. Intriguingly, the Atiyah-Singer index theorem as described by Giesbers *et al* [71] and Katsnelson *et al* [72]. Predicts that the number of zero-energy LL states in graphene is a topological invariant even if the magnetic field is inhomogeneous. Therefore, the number of zero-energy states is only determined by the total magnetic flux, and broadening of the lowest LL is reduced.

**Figure 8.**
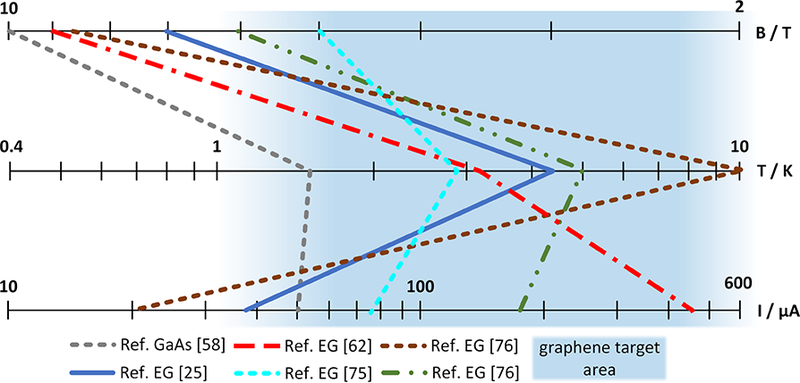
Comparison of typical experimental conditions of GaAs/AlGaAs [58] and graphene QHE samples [25, 62, 75, 76] with high accuracy resistance quantization in the *i* = 2 plateau.

The 2008 report ‘Quantum Resistance Metrology in Graphene’, Giesbers, Rietveld *et al* [73] described the first metrological characterization of the QHE in graphene, finding a Hall resistance *ρ*_*xy*_ equal to *h*/2*e*^2^ to within 15 μΩ Ω^−1^ using a 1 mm wide exfoliated graphene device, limited by low breakdown current (⩽2.5 μA) and excess contact resistance. Figure 8 shows a collection of the most recent published experimental conditions of high quality GaAs/AlGaAs and graphene QHE samples with a relative uncertainty of resistance quantization approaching 10^−9^. There are also additional published measurements (not shown in the diagram) that demonstrate that GaAs/AlGaAs may also be used at significantly higher currents [74] or lower magnetic flux densities [51]. However, pushing the limits of one measurement parameter always needs to be compensated by another parameter such as by decreasing the temperature. The comparison of recent results in figure 8 shows that graphene based QHE samples have a clear overall advantage when going to low magnetic fields, higher temperatures and larger currents that allow measurements using less complex systems.

## 4. Outlook

As efforts in production of EG for electrical resistance metrology continue to move forward, NMI groups have envisioned several goals for the new standard. Presently, NMI resistance scaling with QHR is mainly realized at low bridge voltages of 0.5 V–1 V using room temperature direct current comparator (DCC) and cryogenic current comparator (CCC) bridges. Compact measurement systems using cryogen-free table-top cryostats could provide turn-key resistance traceability and significantly reduce the costs due the relaxed measurement conditions provided by graphene-based Hall devices [75, 77, 78]. Some of these systems already operate using room temperature DCC bridges. One such compact QHR system described by Rigosi *et al* operates using an EG device at 5 T, 2.8 K to 3.0 K, and source-drain current *I*_SD_ ≈ 100 μA, where measurements can provide relative uncertainty below 10^−8^ [75].

The future chain of traceability would benefit by utilizing higher voltages or currents, allowing full compatibility between EG quantum Hall devices and cryogen-free measurements. These development efforts focus on improving the accessibility of quantum resistance standards to the international community of NMIs and industry. One approach is the scaling to other quantised values by using QHE devices in series and parallel connections [79] or by p-n junction arrays [80]. In the traditional approach, multiple individual Hall bar devices are connected in parallel or series to create resistance values of *qR*_K-90_ where *q* is a positive rational number. However, in larger networks, an increasing influence of the contact resist ance and localized inhomogeneous carrier concentration may limit the precision of resistance quantization. The exceptional suitability of EG for this purpose is due to the pinning of the Hall resistance *i* = 2 plateau over a very wide range of magn etic flux densities [13, 14] as well as the symmetrical energy spectrum and repeatable low contact resistance. The giant resistance plateau allows resistance quantization in all sample regions despite local variations in the charge carrier density. Thus, the Delahaye multiple-series connection technique [81] would enable room-temperature bridges to operate with voltages of 10 V or more across a series of devices, allowing greater precision in scaling to high resistance standards of value 1 MΩ or above.

The symmetric energy spectrum of graphene supports conductivity using both holes and electrons in the same device through gating or doping techniques. This is achieved by integrating several Hall elements in one device using alternating p-conducting and n-conducting graphene regions, thus reducing the complexity of network connections [80]. Electric gates deposited above a hexagonal boron nitride dielectric layer have been used to create such a device, which showed good resistance quantization with an accuracy of 0.1 μΩ Ω^−1^ [82]. These results demonstrate the feasibility of p-n junctions for metrology but also show the need of further development with respect to gating or doping techniques that allow for the design of more complex devices that are capable of scaling within a wide range of resistance values with assured high accuracy. Similar implementations of multiple graphene devices have been suggested for use with ac-resistance scaling methods [83].

The major reason for the success of the QHR with direct current (dc) for the realization of the unit ohm is its high robustness and its universality due to its direct relation to the natural constants *h* and *e*. Moreover, NMI research on the QHR with alternating current (ac) opened new routes that enable connecting the ohm to other units such as the farad and henry by natural constants [84, 85]. Compared to GaAs, ac-quantum Hall measurements using epitaxial graphene were demonstrated to be favorable due to improved ac loss characteristics that allow for high precision primary impedance standards and enable direct access to the physical quantities capacitance and inductance [86–88].

In conclusion, as the new SI is implemented in the coming year, EG devices exhibiting the QHE can provide a robust avenue to electrical resistance standards and metrology in general. The current efforts have shown great promise in being able to provide a smooth transition from the use of burdensome infrastructure found with GaAs-based QHR systems to the operation of user-friendly cryocooler systems in many labs.
